# Supplementary material for: Copper Ion Mediates Yeast-to-Hypha Transition in Yarrowia lipolytica
Source: J Fungi (Basel). 2023 Feb 13;9(2):249. doi: 10.3390/jof9020249 (PMC9966917; doi:10.3390/jof9020249)
Supplement: Supplementary file 1 [file jof-09-00249-s001.zip › Figure S1.pdf]

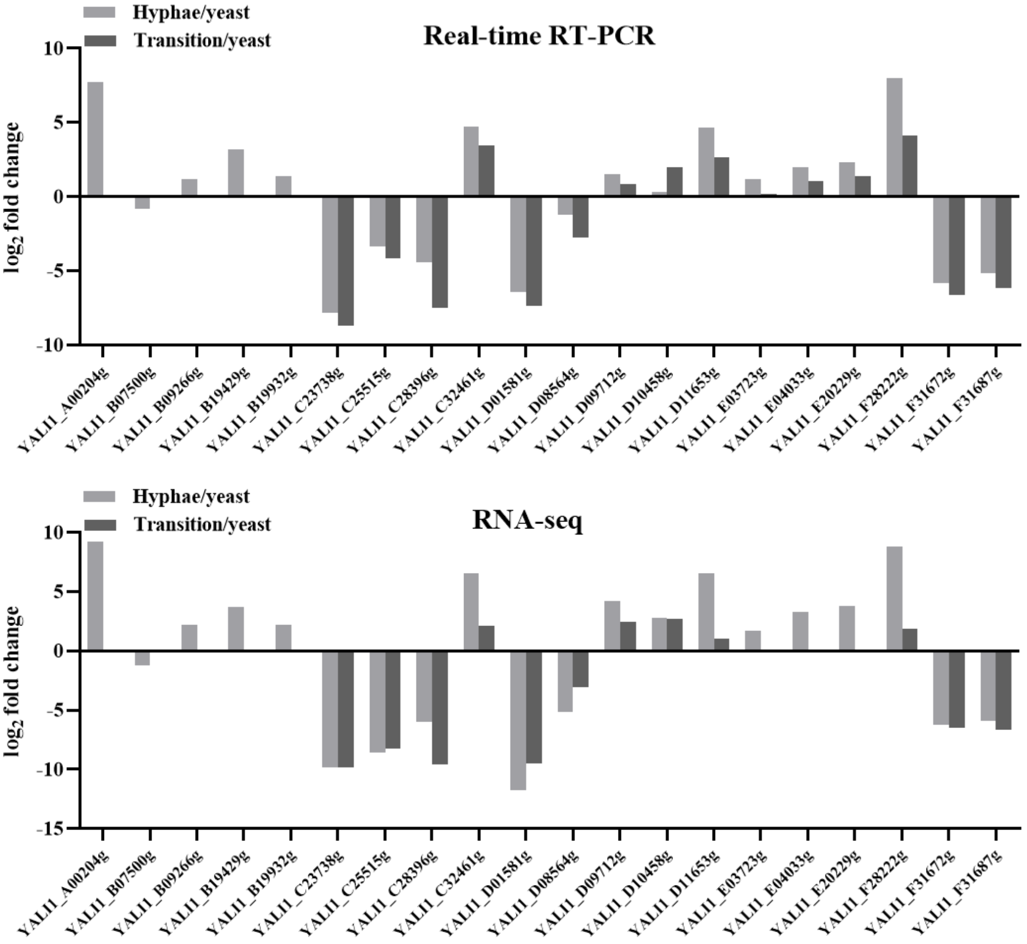

Figure S1. Fluorescent real-time RT-PCR validation of 20 differentially expressed genes in RNA-Seq. *ACT1*(*YALI0D08272g*) was used as the reference gene to normalize the gene expression in fluorescent real-time RT-PCR.
