# Supplementary material for: Copper Ion Mediates Yeast-to-Hypha Transition in Yarrowia lipolytica
Source: J Fungi (Basel). 2023 Feb 13;9(2):249. doi: 10.3390/jof9020249 (PMC9966917; doi:10.3390/jof9020249)
Supplement: Supplementary file 1 [file jof-09-00249-s001.zip › Table S3.pdf]

Table S3. Strains and plasmids used in this study.

Plasmid/Strain	Description	Source
W29	Wild-type *Y. lipolytica* strain	
Po1f	MatA, leu2-270, ura3-302, xpr2-322, axp1-2, Leu− , Ura− , △AEP, △AXP	1
Po1f-TLL	Po1f derivative, carrying a TLL expression cassettes	this laboratory
pUAxp7166-ROL	Data not published	this laboratory
pUAxp7166-D11653g	pUAxp7166-ROL derivative, carrying YALI1_D11653g gene	this study
pCRISPRyl	CRISPR/Cas9 vector for *Y. lipolytica*, with AvrII site for sgRNA insertion	2
pCRISPRyl-mhy1	Po1f derivative, carrying the sgRNA sequence of mhy1	this study
	**Reference**	
1	Madzak C, Tréton B, Blanchin-Roland S. Strong hybrid promoters and integrative expression/secretion vectors for quasi-constitutive expression of heterologous proteins in the yeast *Yarrowia lipolytica*. J Mol Microbiol Biotechnol. 2000 Apr;2(2):207-16.	
2	Schwartz CM, Hussain MS, Blenner M, Wheeldon I. Synthetic RNA Polymerase III Promoters Facilitate High-Efficiency CRISPR-Cas9-Mediated Genome Editing in *Yarrowia lipolytica*. ACS Synth Biol. 2016 Apr 15;5(4):356-9.	
