# Supplementary material for: Copper Ion Mediates Yeast-to-Hypha Transition in Yarrowia lipolytica
Source: J Fungi (Basel). 2023 Feb 13;9(2):249. doi: 10.3390/jof9020249 (PMC9966917; doi:10.3390/jof9020249)
Supplement: Supplementary file 1 [file jof-09-00249-s001.zip › Table S4.pdf]

Table S4. Oligonucleotide sequences of primers used in this study.

Primer	Sequence (5′→3′)a
N-D11653g-F	CACATACAACCACACACATCCACAGCGGCCGCCACCATGGCTCACATCCGCTTTGC
C-D11653g-R	GTGGGGACAGGCCATGGAATCGATCTAATAGATGTAAAAGAACGTGATATGGACCGT
Nsi1-D11653-F	TTGGACGATGATGCTTTGAGGGC
Nsi1-D11653-R	GCCCTCAAAGCATCATCGTCCAA
N-A00204-F	CACATACAACCACACACATCCACAGCGGCCGCCACCATGCCCGACTCTGCTGAACTC
C-A00240-R	GTGGGGACAGGCCATGGAATCGATTCAGTGACAGGAGCGTCGGTA
N-B07500-F	CACATACAACCACACACATCCACAGCGGCCGCCACCATGCCTGACGCTCGACTCT
C-B07500-R	GTGGGGACAGGCCATGGAATCGATCTACTGGAAAAATCGGCATTGCCG
Nsi1-B07500-F	CCTGTGGAATGCACTTCGACAATGT
Nsi1-B07500-R	ACATTGTCGAAGTGCATTCCACAGG
N-B09266g-F	CACATACAACCACACACATCCACAGCGGCCGCCACCATGTCTTGGCAAGGTGAGCAG
C-B09266-R	GTGGGGACAGGCCATGGAATCGATCTAATAGTTAGCATTGATGAGATAATCTCCCAGAGC
N-B17569-F	CACATACAACCACACACATCCACAGCGGCCGCCACCATGTCTGAAAAGGTGAAAATTGAAGCCAAG
C-B17569-R	GTGGGGACAGGCCATGGAATCGATTTACTCATAAAACTTGCGGTCGAGTTCAC
N-B19429g-F	CACATACAACCACACACATCCACAGCGGCCGCCACCATGCAGTTCTCCGCCGC
C-B19429-R	GTGGGGACAGGCCATGGAATCGATTTAGATGAGCTGAGCAAGAGCACC
N-B19932-F	CACATACAACCACACACATCCACAGCGGCCGCCACCATGGTCGCCACTGTTCAGC
C-B19932-R	GTGGGGACAGGCCATGGAATCGATTTATTTGTTGGCCTTCTCGAAGTACTCC
N-C08017g-F	CACATACAACCACACACATCCACAGCGGCCGCCACCATGTCCTACACCCGAGTCACT
C-C08017-R	GTGGGGACAGGCCATGGAATCGATCTACAGCTTGCTGTCCAGCTTG
N-C12900-F	CACATACAACCACACACATCCACAGCGGCCGCCACCATGTCTGTCTCCAAGGTGAGTATTTGAAG
C-C12900-R	GTGGGGACAGGCCATGGAATCGATTTAGGCGCCAAGGGCG
N-C18396-F	CACATACAACCACACACATCCACAGCGGCCGCCACCATGTACAACAACACACGGGGG
C-C18396-R	GTGGGGACAGGCCATGGAATCGATTCAACACCATTGCCTCCACAC
N-C23738-F	CACATACAACCACACACATCCACAGCGGCCGCCACCATGCACGGTCTCAATCAGTCAGCA
C-C23738-R	GTGGGGACAGGCCATGGAATCGATTTAGTACTTGGCCAGAGGTCGCTG
N-C25515-F	CACATACAACCACACACATCCACAGCGGCCGCCACCATGGGCAATTTTTGCCCGTCC
C-C25515-R	GTGGGGACAGGCCATGGAATCGATCTAATGCTGAGCCTCAGGACGAAT
N-C27382g-F	CACATACAACCACACACATCCACAGCGGCCGCCACCATGAGCAAGCGCCAGAATGAC
C-C27382g-R	GTGGGGACAGGCCATGGAATCGATCTATTTCAACTGGCTCTTGAGCCC
C27382g-Nsi1-F	GACATAGCATGTATGGTGCTTGAC
C27382-Nsi1-R	GTCAAGCACCATACATGCTATGTC
N-C28396g-F	CACATACAACCACACACATCCACAGCGGCCGCCACCATGCACATTTCACTAATCGCAATCAC
C-C28396-R	GTGGGGACAGGCCATGGAATCGATCTAACAGCAACAATCCTCGCCC
N-C32461-F	CACATACAACCACACACATCCACAGCGGCCGCCACCATGACAACCTTCTATCTCGGCTACT
C-C32461-R	GTGGGGACAGGCCATGGAATCGATTCAAAAGCTGCGAGACAGCCG
N-D01548-F	CACATACAACCACACACATCCACAGCGGCCGCCACCATGGCGCGCAAAACGGAC
C-D01548-R	GTGGGGACAGGCCATGGAATCGATCTACATAATCACTTGATTCCAGCCCCT
Nsi1-D01548-F	CACAAACACCATGCTTCAATGTCCA
Nsi1-D01548-R	TGGACATTGAAGCATGGTGTTTGTG
N-D01581g-F	CACATACAACCACACACATCCACAGCGGCCGCCACCTTAATTCCACCCAATGACTCCATCTCC
C-D01581g-R	GTGGGGACAGGCCATGGAATCGATATGTGCGGACCCTCAAGAAAC

N-D08564g-F	CACATACAACCACACACATCCACAGCGGCCGCCACCATGGCCAACGCGTTTTCC
C-D08564-R	GTGGGGACAGGCCATGGAATCGATTTAGGCAACCTCCTTTCGTTCG
N-D09712g-F	CACATACAACCACACACATCCACAGCGGCCGCCACCATGTTTCCGTGTCTGGTTGCC
C-D09712-R	GTGGGGACAGGCCATGGAATCGATCTATACATTACAAATTAGTGCATGGTGCTTTTC
N-D10458g-F	CACATACAACCACACACATCCACAGCGGCCGCCACCATGTCCAAAGCAGTAGGAATTGATCTTGG
C-D10458-R	GTGGGGACAGGCCATGGAATCGATTTAGTCAACCTCCTCAACGGTGG
N-E03723-F	CACATACAACCACACACATCCACAGCGGCCGCCACCATGAACGTCATATTCGAAAACACGCC
C-E03723-R	GTGGGGACAGGCCATGGAATCGATTCAACGGTTTTCGAGGACCG
N-E04033g-F	CACATACAACCACACACATCCACAGCGGCCGCCACCATGGCACCACTAACAAAGTCGC
C-E04033-R	GTGGGGACAGGCCATGGAATCGATCTACTGGGCCAGAATGGACTTGAC
N-E20229g-F	CACATACAACCACACACATCCACAGCGGCCGCCACCATGAACTTCGACTATGACTTTCTGCAGT
C-E20229-R	GTGGGGACAGGCCATGGAATCGATCTAATGTCCTTCGAAGTCCGGTGAAG
N-E34205-F	CACATACAACCACACACATCCACAGCGGCCGCCACCATGGCTGAAGATAAGGTGTTTGAACTGG
C-E34205-R	GTGGGGACAGGCCATGGAATCGATTTAGAAATCGTCCTCATCCACGATGAAC
N-E36937-F	CACATACAACCACACACATCCACAGCGGCCGCCACCATGAAACGAACTCGAGTGGTCAAG
C-E36937-R	GTGGGGACAGGCCATGGAATCGATTCAATCTTCACTCTTGGCCAGC
Nsi1-E36937-1F	CCAGAGGAACATGCTTTCAAGGTC
Nsi1-E36937-1R	GACCTTGAAAGCATGTTCCTCTGG
Nsi1-E36937-2F	GAAATCCCCATGCACATGCGC
Nsi1-E36937-2R	GCGCATGTGCATGGGGATTTC
N-E37419-F	CACATACAACCACACACATCCACAGCGGCCGCCACCATGATTCTCGTCCAGGGCGAAA
C-E37419-R	GTGGGGACAGGCCATGGAATCGATCTACTCCCAAGTAAACCTCTCCCCT
Nsi1-E37419-F	CCGAGTCGTATGCACGGAAATGGA
Nsi1-E37419-R	TCCATTTCCGTGCATACGACTCGG
N-F06904g-F	CACATACAACCACACACATCCACAGCGGCCGCCACCATGAAAAACGTCATTTACTTTTGCAAGTGAGT
C-F06904-R	GTGGGGACAGGCCATGGAATCGATTCAATTGTTAATGTTTCTCCACTCGGACT
N-F17805-F	CACATACAACCACACACATCCACAGCGGCCGCCACCATGGACCATCTCTCTCAAGCAGG
C-F17805-R	GTGGGGACAGGCCATGGAATCGATCTAGAAAATGACACTCTTGCCACTCTG
N-F28222g-F	CACATACAACCACACACATCCACAGCGGCCGCCACCATGGAATCGTCGGGTATCTGTCG
C-F28222g-R	GTGGGGACAGGCCATGGAATCGATTCAGCCAGAGTTTACTCGCGAG
N-F29317-F	CACATACAACCACACACATCCACAGCGGCCGCCACCATGAGTACCCCTCCTTATTACAACATCTACC
C-F29317-R	GTGGGGACAGGCCATGGAATCGATTTACGCATCACCGGCCT
N-F31672g-F	CACATACAACCACACACATCCACAGCGGCCGCCACCATGCAGCTCTCAGCCCTCG
C-F31672-R	GTGGGGACAGGCCATGGAATCGATTTAGATGAGGAGGGCGGCG
N-F31687-F	CACATACAACCACACACATCCACAGCGGCCGCCACCCTAGCTTGCGTAACGGGCA
C-F31687g-R	GTGGGGACAGGCCATGGAATCGATATGAGCCACGACCACGG
N-F39789-F	CACATACAACCACACACATCCACAGCGGCCGCCACCATGCGCCTGGAGCTGC
C-F39789-R	GTGGGGACAGGCCATGGAATCGATTCAGTGTCTAGGTCGGTGCATGG
CX7166-F	GATCTACTGATCACGGGCAAAAGTGC
CX7166-R	GGTGGTGGGAAGAACACCG
T-R	GATAAGCTGTCAAACATGAGAATTCGG
mhy1-CX-F	TACCACGCTTTCCCCACGAC
mhy1-CX-R	AGATGGAAACGGCGTCCTTCTC
ver-mhy1-R	AATTCCCGTCTTGCATTCCA

verF	CCCCAGTTGCAAAAGTTGACAC
mhy1-sgF	GGGTCGGCGCAGGTTGACGTCACCCTTCGAGGCCAGATGT
mhy1-sgR	GCTATTTCTAGCTCTAAAACACATCTGGCCTCGAAGGGTG
