# Supplementary material for: Copper Ion Mediates Yeast-to-Hypha Transition in Yarrowia lipolytica
Source: J Fungi (Basel). 2023 Feb 13;9(2):249. doi: 10.3390/jof9020249 (PMC9966917; doi:10.3390/jof9020249)
Supplement: Supplementary file 1 [file jof-09-00249-s001.zip › Table S5.pdf]

Table S5. Primers used for Fluorescent real-time RT-PCR in this study.

| Primer | Sequence (5′→3′)a |
|---|---|
| A00204-F | AACGTAGCTACCCTTCTGGAAC |
| A00204-R | CATTCTCATCCAGCCAAGCTTC |
| B07500-F | ATGACCAAGGAGCATCAGGATG |
| B07500-R | GCCGTTTCGATCCTTCTTCTTG |
| B09266-F | GTTGACAACCTGGTTGGATCTG |
| B09266-R | GAAGTACTTCTGGCCAGCAATG |
| B17569-F | TACAGCAGATTGTCACGTCCAA |
| B17569-R | CTTGTCTTCGAGCTCACTGACT |
| B19429-F | CAGAAGACTCGGCCTTGGAAG |
| B19429-R | CTTCTTCTGCTGTCATTGGCTC |
| B19932-F | CCAAGGACTACGGTGTTCTCAT |
| B19932-R | TCTCAGTGAACTGGAAGGCATC |
| B21538-F | TGGAATGAAGCTAACGGAACCA |
| B21538-R | GATGAATCCGACCACAGCAATG |
| C08017-F | GTTGCTGGCGGAATTAAGTCTG |
| C08017-R | TTACCACCGGCAATACCAATGA |
| C12900-F | GAGTACCTTGCCGAGCAGAAG |
| C12900-R | AATCTCGAGCAGAACCTTCTCC |
| C18396-F | TCGATATGGCAGACACAAGGAC |
| C18396-R | GCTGAGACTGACCTCGAGTATG |
| C23738-F | GGTCAGACAAATTGCTCTGCAG |
| C23738-R | CTTGGCAATCTCCTCCTTGAGT |
| C25515-F | GCTAAGCGCACGTATCTCTCTA |
| C25515-R | ACCAGAAACCGGACACATTGTA |
| C27382-F | TGAGTGAGGATGAGGAGGGAAT |
| C27382-R | CCAATCGAGTCTCCAAGACCTC |
| C28396-F | GACTGGCCCTTCTTAGACAGAC |
| C28396-R | GCAACAGTCACTCGGATTAGGT |
| C32461-F | CTGCAACCATCAAGGCTCTAGA |
| C32461-R | GAAGGTGTTACTGCAAGGGAGA |
| D01548-F | CAGATTGACTTCTTCCTGGGCT |
| D01548-R | CTGTCCACCTGTATGACACTCC |
| D01581-F | TGGGATCCACTTCAGACACAAG |
| D01581-R | TCTCAGTTACAAAGTCGAGCCC |
| D08564-F | CTACTCGCTGGACAAGGATCTC |
| D08564-R | GGGTCTTGGCAATCTTGATTCG |
| D09712-F | TTGCCCAGAAGAACGGTACC |
| D09712-R | CGTTGGCAATGGAGTAGGAGG |
| D10458-F | AGATCACCATCACCAACGACAA |
| D10458-R | TGAGAGAGTAGGCGTAGGACTC |
| D11653-F | CGGAAGCGAACGAATCTACCAA |

| | |
|---|---|
| D11653-R | ATGCAGACTTGGATACCGAGGA |
| E03723-F | TAGTGATCTTCATGTACGCGCA |
| E03723-R | TACAGCAGCATCAAATCCCTGT |
| E04033-F | ACCCTCGCTGAGATTGATGATC |
| E04033-R | TCCTTGTCGAACACCTGGAATC |
| E20229-F | GAAGACGGCAAGTTTCTGTGTC |
| E20229-R | TCTTGAGATCGTGCTGTCTGAC |
| E34205-F | ATTGATATCCGGGCCTTCTACG |
| E34205-R | GCATCTTGGATGCTTTGCACTT |
| E36937-F | TGTGACTCGAAAGGTGAATCGT |
| E36937-R | TCCAGTAGAACCAGCAGTTGAC |
| E37419-F | CTGCTGCACATATCAACCACAG |
| E37419-R | CGGCTGCTGGTAATAACTGTTG |
| F29317-F | CATAGGAACAGCTCTCACGGAG |
| F29317-R | TAGGTATGCAATTCGCCCTTGA |
| F06904-F | ACTATAAGAAGCTTCCTCGGCC |
| F06904-R | CGACTTGAGCTTGACGACATTC |
| F17805-F | TCGCTGGTCATGAAGAACATCA |
| F17805-R | CAGATTGACGCTCATTGGACAC |
| F28222-F | TTGAGGTCGTCAATCAGAGTGG |
| F28222-R | ACCCTTGACATCATCACTCACC |
| F31672-F | CAAGATCGGTGAGCGATTCAAG |
| F31672-R | GCCGTAAAGCACCTTACTGAAG |
| F31687-F | GAGCTCATCCTCGATATCTCGC |
| F31687-R | GAAGCTCATCAAGTGTGGCAAG |
| F39789-F | CCTCATGACACACCACAACAAC |
| F39789-R | GACCATCACACAGAGTCACCAA |
| ACT-F | CTGGCCGAGATCTTACCGAC |
| ACT-R | CATCGGGAAGCTCGTAGGAC |
